# Characteristics of a *Dinophysis* cf *acuminata* Population from a Tidewater Glacier Lagoon in a Temperate Latitude: Applications to *Dinophysis* Studies

**DOI:** 10.3390/md24030096

**Published:** 2026-02-28

**Authors:** Patricio A. Díaz, María García-Portela, Gonzalo Álvarez, Francisco Rodríguez, Iván Pérez-Santos, Daniel Varela, Michael Araya, Camila Schwerter, Ángela M. Baldrich, Barbara Cantarero, Beatriz Reguera

**Affiliations:** 1 Centro i~mar, Universidad de Los Lagos, Casilla 557, Puerto Montt 5480000, Chile; patricio.diaz@ulagos.cl (P.A.D.); ivan.perez@ulagos.cl (I.P.-S.); dvarela@ulagos.cl (D.V.); camilaschwerter1@gmail.com (C.S.); angela.baldrich@ulagos.cl (Á.M.B.); 2 Centro Interdisciplinario para la Investigación Acuícola (INCAR), Universidad de Concepción, Concepción 3349001, Chile; 3 Centro Oceanográfico de Vigo (IEO-CSIC), Subida a Radio Faro 50, 36390 Vigo, Spain; maria.garcia@ieo.csic.es (M.G.-P.); francisco.rodriguez@ieo.csic.es (F.R.); 4 Departamento de Acuicultura, Facultad de Ciencias del Mar, Universidad Católica del Norte, Coquimbo 1780000, Chile; gmalvarez@ucn.cl; 5 Centro de Investigación y Desarrollo Tecnológico en Algas (CIDTA), Facultad de Ciencias del Mar, Universidad Católica del Norte, Coquimbo 1780000, Chile; mmaraya@ucn.cl; 6 Center for Ecology and Sustainable Management of Oceanic Islands (ESMOI), Departamento de Biología Marina, Facultad de Ciencias del Mar, Universidad Católica del Norte, Coquimbo 1780000, Chile; 7 Centro de Investigación Oceanográfica COPAS Coastal, Universidad de Concepción, Concepción 3349001, Chile; 8 Programa de Doctorado en Biología y Ecología Aplicada, Universidad Católica del Norte, Coquimbo 1780000, Chile; barbarapazcf@gmail.com

**Keywords:** *Dinophysis acuminata*, tide water glacier lagoons, San Rafael Lagoon, northwestern Patagonia, Chile

## Abstract

*Dinophysis acuminata*, the main agent of diarrhetic shellfish poisoning (DSP) worldwide, shows a high variability in morphology and toxin content between strains from contrasting habitats. Most frequent uncertainties in morphological discrimination are within the “*D. acuminata* complex”, but confusion with other species (e.g., *D. norvegica*, *D. fortii*) also occurs. Here we describe a unique PTX2-containing population of *Dinophysis* cf *acuminata* observed during opportunistic samplings in San Rafael Lagoon (Chilean Patagonia), the only tidewater glacier lagoon remaining in the glacier with the world’s lowest latitude. *Dinophysis acuminata* was the only *Dinophysis* species observed during three seasonal surveys in the well-mixed cold (4–7° C) and brackish (salinity 14–15) waters of the lagoon. Cell densities ranged from 500 cells L^−1^ (winter) to 2800 cells L^−1^ (summer). Partial sequences of their ITS rDNA aligned them with *D. acuminata* strains from Europe and North America, and sequences of their stolen plastids 23S rDNA confirmed ciliates of the *Mesodinium rubrum* + *major* complex as their prey and plastid source. All these reasons make this lagoon a highly sensitive area and natural laboratory for climate change-related topics and *Dinophysis* issues related to (i) the effect of long-term exposure of marine fauna to pectenotoxins and (ii) the adaptations of *D.* cf *acuminata* to persist in a unique ecosystem with austral water characteristics located in a warm temperate latitude light regime. Results here add knowledge to the biogeography and habitat ranges of *D. acuminata* and the problems faced to monitor and provide early warning of its distribution.

## 1. Introduction

Among a variety of harmful algal blooms (HABs) and impacts, blooms of toxin-producing microalgae (toxic HABs) are the most damaging to public health (human poisonings) as well as to the shellfish (harvesting closures), fish (mass mortalities) and aquaculture industries. Some microalgae produce potent toxins which even at low cell concentrations are accumulated and transformed by filter feeders and transferred through food webs [1,2]. This “particulate” transmission of toxins was formerly considered the only way harm was caused during paralytic (PSP) and diarrhetic shellfish poisoning (DSP) events. Evidence has accumulated in the last 20 years of extracellular excretion or leakage of toxins into the environment [3,4]. In laboratory experiments, the extracellular fraction may comprise 65–90% of the total toxin produced by *Dinophysis* [5,6]. In other words, measuring toxins in particulate form underestimates the real amount of toxins produced and the potential to cause harm to marine organisms [7,8]. 

MacKenzie et al. [9] pioneered the use of passive samplers or “SPATT” (solid-phase adsorption toxin-tracker) resins which adsorb the “dissolved toxins” fraction, dissolved meaning here the fraction of toxins which are not retained after filtering seawater through 0.22 µm pore-sized filters. These toxins are actively released or simply leak into the environment and when found free outside the cells (detectable using the appropriate resin sampler) can affect a much larger range of organisms, from protists to invertebrates [8]. They can also adhere to organic debris and sediment and be detected in plankton concentrates when the toxic cells are no longer present; they may also become available to bottom-dwelling detritivorous invertebrates and browsers [4,10].

Accumulation of toxins in bivalve mollusks (cultures and shellfish beds) exposed to endemic blooms of *Dinophysis* represents the main threat to sustainable exploitation of shellfish resources in Europe [11] and northeastern Japan [12,13] and is an emergent hazard in the US [14]. Diarrhetic shellfish toxins affect consumers of shellfish collected without sanitary control and cause lengthy harvesting bans whenever toxins in shellfish flesh exceed safe limits enforced by regulations [15].

The genus *Dinophysis* Ehrenberg (1841) comprises over a hundred species worldwide [16], of which 10 have been associated with shellfish poisoning [15]. Toxic species of *Dinophysis* produce one or two groups of lipophilic toxins: (i) okadaic acid and derivatives (the diarrhetic shellfish toxins, DSP) and (ii) pectenotoxins [15]. Pectenotoxins (PTXs) were traditionally included with okadaates in the DSP complex because they are both produced by *Dinophysis* and are coextracted with lipophilic solvents used for standard toxin tests [17]. Okadaic acid and derivatives (Dinophysistoxins) are protein-phosphatase inhibitors and the only ones with diarrhetic effects [18,19]. Pectenotoxins may kill mice when administered via intraperitoneal injection and cause hepatotoxicity but are harmless by oral intake [20,21]. Based on recent toxicological studies, the World Health Organization consider that PTXs do not pose a risk to human health, and this group of toxins is no longer under regulation. Nevertheless, pectenotoxins are actin-polymerase inhibitors which affect cellular motility [22], and there is a growing number of studies in vitro about the noxious effects of PTXs in early larval stages of bivalves and fish [23,24]. Concerning subacute effects on human health, okadaic acid and derivatives are considered to be tumour promoters [25], while PTX2 has been found to have a selective apoptotic effect on carcinogenic cells [26,27]. Thus, the same organisms (*Dinophysis* species) produce both tumour promoters and suppressors.

*Dinophysis* species, in particular those belonging to the “*Dinophysis acuminata* complex”, are endemic in many coastal areas with relevant shellfish resources [15]. Monitoring *Dinophysis* spp. may be difficult for various reasons. First, they develop low biomass blooms. Concentrations of a few hundred cells per litre have been associated with toxicity in shellfish, and cell numbers above 10^3^ cell L^−1^ are considered a bloom [17,28], but they can be present most of the year below the detection levels (40–100 cells L^−1^) of traditional monitoring programmes. Second, they show a high intraspecific morphological and toxinological variability, i.e., differences in size and shape and in toxin profile and content can be higher between strains of the same species than between different species from the same location. Lastly, there is low inter-species variability in the nuclear (LSU, SSU and ITS regions) and mitochondrial gene (*cox1* and *cob*) sequences normally used to discriminate between morphologically close species and applied in the design of molecular probes for their identification [29,30,31,32]. Considering that the morphological characteristics commonly used to diagnose *Dinophysis* species are the shape, relative size, and ornamentation of the large hypothecal plates [33,34,35], species identification can be cumbersome when morphologically similar species, each one with their array of morphotypes, co-occur in the same sample, and molecular probes do not help much to solve the uncertainties.

Key issues to predict toxic blooms of *Dinophysis* species and the subsequent contamination of shellfish are related to the identification of their nutritional sources (ciliate prey) and life history strategies (overwintering cells and origin of the *inoculum* or “population source”) [36].

*Dinophysis* species are “plastidic specialist non-constitutive mixotrophs” (pSNCM), i.e., their plastids are not constitutive, but stolen (kleptoplastids) from very selected prey (specialists) [37]. A three-species food chain—*D. acuminata* fed the ciliate *Mesodinium rubrum*, the later fed cryptophyte microflagellates—was established in laboratory cultures two decades ago [38] and later used with other toxic *Dinophysis* (*D. acuta*, *D. caudata*, *D. fortii*, *D. infundibulus*, *D. norvegica*, *D. sacculus* and *D. tripos*) [39,40]. However, there are records of field populations of *Dinophysis* with kleptoplastids outside the *Teleaulax*/*Geminigera* clade which suggest the possibility of alternative prey [41,42,43]. Given the mixotrophic nature of *Dinophysis* species, it is crucial to add its confirmed ciliate prey (*Mesodinium rubrum* + *major* complex or any other suspected plastid-bearing ciliate) to the growth equation for a sound bloom prediction model. But so far, few *Dinophysis*-focused surveys have included analyses of the accompanying potential prey (plastid bearing microciliates) nor of the plastid sequences of both, predator and suspected prey, to confirm their common cryptophyte origin [44,45,46,47,48].

In addition, unlike other HAB dinoflagellate species (e.g., *Alexandrium* and *Protoceratium*), *Dinophysis* species do not seem to rely on benthic stages (sexual cysts) for seeding. Their complex polymorphic life cycle includes sexual processes and formation of planozygotes. These mobile diploid planozygotes can divide to produce vegetative cells, short-cutting the sexual-cyst stage [49]. Thus, the whole life cycle comprises free-swimming motile forms (holoplanktonic life cycle) which occur in the water column; the next season *inoculum* will be formed by free mobile forms which overwintered in some quiescent state, dispersed in the water column or in some unknown retention area acting as a “pelagic seed bank”. This term was coined by Smayda [50] to describe the accumulation of dinoflagellates in upwelling fronts like “pelagic seeds” able to act as inocula for future populations when advected to the coast during downwelling.

**Figure 1:**
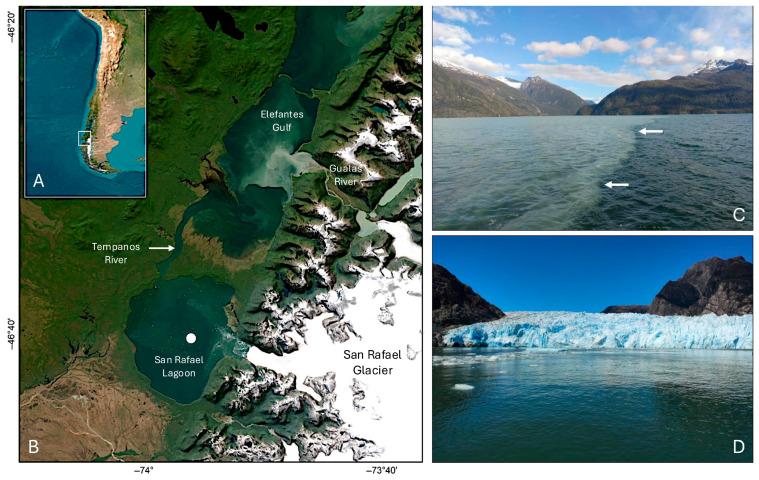
Map showing: (**A**) Chile. Box delimits the Northern Patagonia, Aysén province; (**B**) San Rafael Glacier National Park comprising the Northern Patagonian Ice Field, San Rafael Lagoon, and location of the sampling station, Témpanos “River” channel and the “Elephants Gulf”; photographs of the (**C**) Frontogenesis in Elefantes Gulf (white arrows mark the salinity front and (**D**) San Rafael Glacier Lagoon.

This paper describes a singular population of *Dinophysis acuminata* found in San Rafael Lagoon, a remote relatively unexplored tide-water glacier lagoon of pristine waters and spectacular nature (Figure 1). San Rafael Lagoon, site of a reduced colony of leopard seals (*Hydrurga leptonyx*) and other protected fauna, is part of the San Rafael Glacier National Park [51], designated a World Biosphere Reserve by UNESCO in 1979. This national park, located in the southernmost sector of Northwestern Chilean Patagonia [52], i.e., in a temperate latitude, is site of the Northern Patagonian Ice Field (3500 km^2^), the third largest in the southern hemisphere, after Antarctica and the Southern Patagonian ice fields. These features make San Rafael Park and its water bodies a highly sensitive observatory for the effect of climate change in glacier dynamics [53,54]. Nevertheless, there have been only a few attempts to study the plankton communities (mainly zooplankton) of San Rafael Lagoon [55], an extreme environment formed by the retreat of its homonymous glacier. There is only one survey in the early 1990s, during the international Raleigh Expedition, describing a low diversity net-phytoplankton community comprised by a few diatoms species [56].

*Dinophysis* specimens described here are from opportunistic sampling carried out during seasonal oceanographic surveys to study the hydrography and water mass circulation of the lagoon and adjacent water bodies. In the framework of a One Health approach to the study of toxic algal blooms, the one-way effect of toxins in humans is not the only harm to be considered. The remoteness of San Rafael Lagoon, with neither aquaculture nor exploited shellfish beds, the absence of anthropogenic pollution, stable environmental conditions, and the occurrence during different season surveys (including winter) of only one species of *Dinophysis*, *Dinophysis* cf *acuminata*, make it a natural mesocosm for ecophysiology and population dynamic studies of a cosmopolitan *Dinophysis* species. It can be taken as a model case study to explore the effect of bioactive (particulate or extracellular) compounds of *Dinophysis* on a low diversity local fauna permanently exposed to a single lipophilic toxin producer under stable environmental conditions.

## 2. Results

### 2.1. Hydrographic Conditions and Dinophysis Distribution

**Figure 2:**
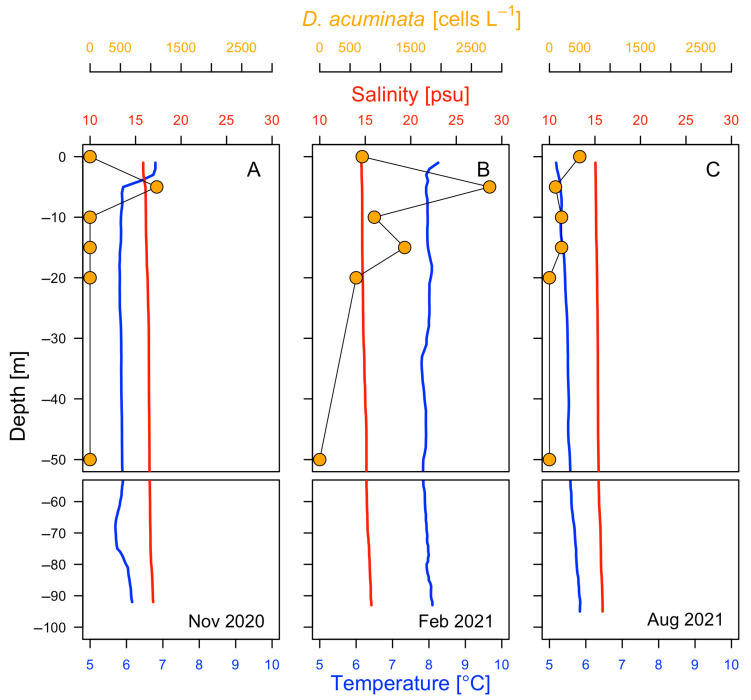
Vertical profiles of temperature (°C), salinity (CTD casts) and cell density of *Dinophysis acuminata* (cells L^−1^) during (**A**) Spring, (**B**) Summer and (**C**) Winter surveys in San Rafael Lagoon (Aysén, Chile).

Vertical distribution of physical properties was measured with CTD casts in spring (November 2020), summer (February 2021) and winter (August 2021). There were light differences in temperature (T) and salinity (S) between surface and bottom (>90 m) waters in summer (T: 8.26 °C and S: 14.56 at the surface; 8.09 °C and 15.67 at 93 m) and winter (T: 5.18 °C and S: 15.5 at surface; 5.84 °C, 15.84 at 95 m). In spring, there was a mild thermal gradient between 0 m (6.8 °C) and 5 m (6.0 °C), and a uniform value from 5 m (6.0 °C) to 92 m (6.16 °C). In winter, there was a very mild thermal inversion from >70 m depth to the bottom (Figure 2). Thus, the lagoon offers a cold (5.2 to 8.3 °C) and brackish (14.6–16.9) but very stable environment with narrow ranges of variability.

*Dinophysis* cf *acuminata* was the only species of *Dinophysis* present in samples collected during the three seasonal surveys. Cells occurrence in winter (500 ± 445 cells L^−1^) at the surface was well above the detection limits in conventional monitoring programmes using microscopic counts following Utermöhl [57] (40–100 cells L^−1^). Moderate bloom levels occurred in spring (2800 ± 980 cells L^−1^) and summer (1000 ± 630 cells L^−1^). Vertical distribution of *Dinophysis* cells varied seasonally; cell maxima were found at the surface in winter and in a sharp maximum at 5 m in spring and summer. This 5 m depth coincided with the base of a weak (maybe diurnal) thermocline observed in the CTD profiles (Figure 2). Low vertical resolution sampling (bottles every 5 m) precluded the possibility to explore the vertical microstructure and determine if *Dinophyis* cells were actively aggregated in a thin layer. It is not certain that the observed peak in *Dinophysis* cells coincided with the maximal intensity of a hypothetical thin layer.

### 2.2. Morphological Observations

**Figure 3:**
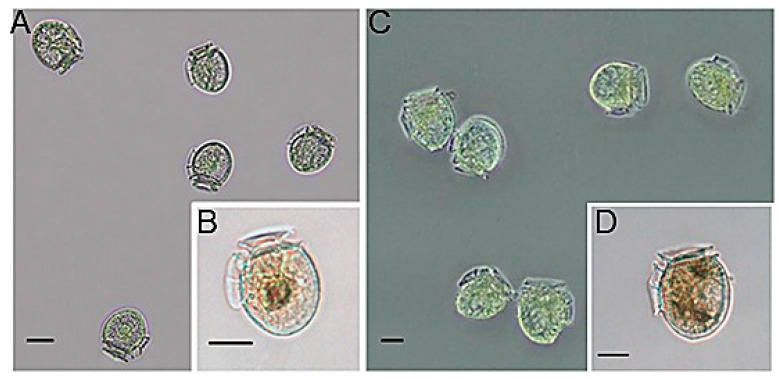
Light micrographs of Lugol’s preserved (**A**,**B**) small and (**C**,**D**) vegetative *Dinophysis* cf *acuminata* cells from San Rafael Glacier Lagoon in late February 2021. Scale bar = 20 μm in all frames.

The micrographs shown here are from summer (February) samples, the only season with a relevant occurrence of the small forms. *Dinophysis* specimens observed in San Rafael Lagoon exhibited a quite regular and constant shape (contour of the large hypothecal plates) and two distinct size-classes or morphotypes during the three seasonal (spring, summer and winter) samplings (Figure 3). The large forms were subovate and slightly asymmetric, and the large hypothecal plates were well ornamented and had a coarse appearance which resembled that of the cold temperate species *Dinophysis norvegica*. Cell measurements were L (length) = 43.53 ± 2.32 µm and D (dorso-ventral depth) = 39.03 ± 2.32 µm, *n* = 44; the L:D ratio was 1.12 ± 0.05. The smaller forms were more oval and symmetric, and measurements were L = 27.68 ± 3.41 µm and D = 25.90 ± 3.65 µm wide, *n* = 9; the L:D ratio was 1.07 ± 0.08 (Figure 3).

### 2.3. General Alignment of 23S rDNA Sequences and Phylogenetic Analyses

The alignment of the plastid-derived sequences of 23S rDNA comprised 43 sequences with a total aligned length of 381 bp (target sequences in this study, 2_23S_San Rafael_*Dinophysis* PX498283 and 6_23S_San Rafael_*Dinophysis* PX498284 were 312 and 306 bp long, respectively). The dataset included 55 parsimony-informative sites, 49 singleton sites, and 277 constant sites. ModelFinder selected the TPM3u + G4 model as the best-fit substitution model according to the Bayesian Information Criterion (BIC). The maximum log-likelihood value obtained for the final tree was −1533.570.

**Figure 4:**
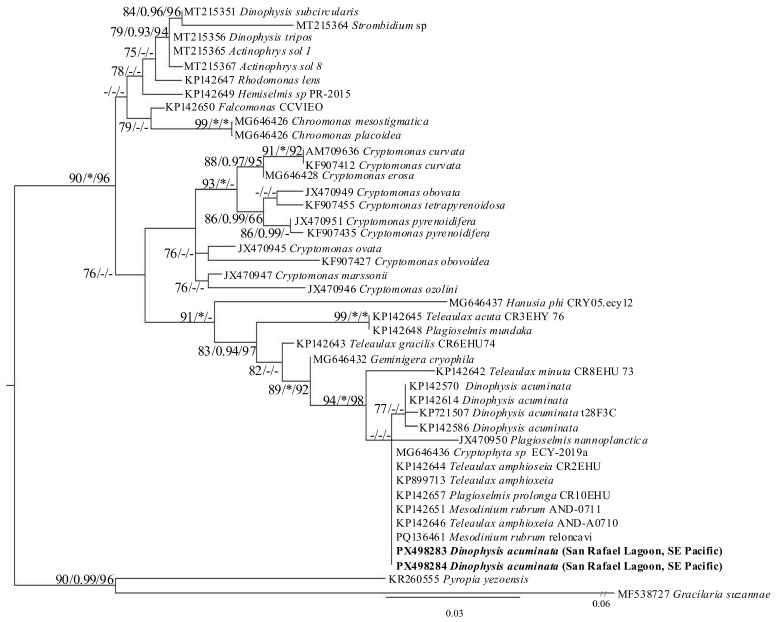
Maximum likelihood phylogenetic tree inferred from 23S rDNA sequences, including sequences of *Dinophysis* species plastids. *Pyropia yezoensis* and *Gracilaria suzannae* were used as outgroups. The 23S sequences of *Dinophysis* cells obtained in this study are in bold. The scale bar represents inferred evolutionary distance in changes/site. Node support represents Shimodaira–Hasegawa approximate likelihood ratio test (SH-aLRT), approximate Bayesian (aBayes) and ultrafast bootstrap (UFBoot) values. Only the values ≥ 70%/≥0.90/≥90% (SH-aLRT/aBayes/UFBoot, respectively) are shown. Asterisks represent maximum statistical support (100%/1/100%).

The resulting maximum-likelihood (ML) phylogeny recovered the main cryptophyte lineages, including *Teleaulax*, *Plagioselmis*, *Geminigera*, *Cryptomonas*, *Chroomonas*, *Rhodomonas*, and *Hemiselmis*. As these sequences are plastid-derived, the resulting phylogeny reflects the cryptophyte origin of the kleptoplastids retained by *Dinophysis* cells rather than the phylogenetic position of the *Dinophysis* host itself. The two plastid-derived sequences obtained from *Dinophysis* (SH-aLRT/aBayes/UFBoot = 91.1/1/86) clustered within the *Teleaulax*–*Plagioselmis-Geminigera* assemblage (e.g., MG646432, KP142648, KP142644, KP142657, KP142646, KP899713). The two sequences obtained in this study were grouped in a subclade (94.1/1.00/98) together with *T. amphioxeia* and *Plagioselmis* sequences. Both *Dinophysis*-derived 23S rDNA sequences showed very high similarity to these cryptophyte sequences: 2_23S_San_Rafael_*Dinophysis* was identical (100%) to *Teleaulax amphioxeia* (KP142644 and KP899713) and *Plagioselmis prolonga* (KP142657), while 6_23S_San_Rafael_*Dinophysis* shared 98.7% identity with the same sequences. These results confirm that the plastids in *Dinophysis* cells from San Rafael belong to a *Teleaulax*/*Plagioselmis*-type cryptophyte lineage (Figure 4).

### 2.4. General Alignment of ITS rDNA Sequences and Phylogenetic Analyses

A total of 71 ITS rDNA sequences were included in the final alignment, which had a length of 646 bp. ModelFinder selected TIM2 + F + G4 as the best-fitting nucleotide substitution model according to the BIC. The ML tree inferred under this model had a log-likelihood of –3560.7830. The seven ITS sequences obtained in this study were highly similar and clustered within the *Dinophysis acuminata* complex clade.

Individual untrimmed sequence lengths ranged from 451 to 560 bp (PX494338 D_ac_8: 512 bp; PX494339 D_ac_9: 451 bp; PX494340 D_ac_R6: 534 bp; PX494341 D_ac_R8: 513 bp; PX494342 D_ac_S1: 514 bp; PX494343 D_ac_S2: 560 bp: PX494344 D_ac_S3: 548 bp), based on nucleotide positions excluding gaps. Pairwise nucleotide identities among the seven sequences ranged from 99.56% to 100%**,** with at most 2 nt differences between any pair of sequences. ML patristic distances were likewise very small (from 3 × 10^−6^ to 0.00563 substitutions per site), confirming extremely low ITS divergence within the group.

**Figure 5:**
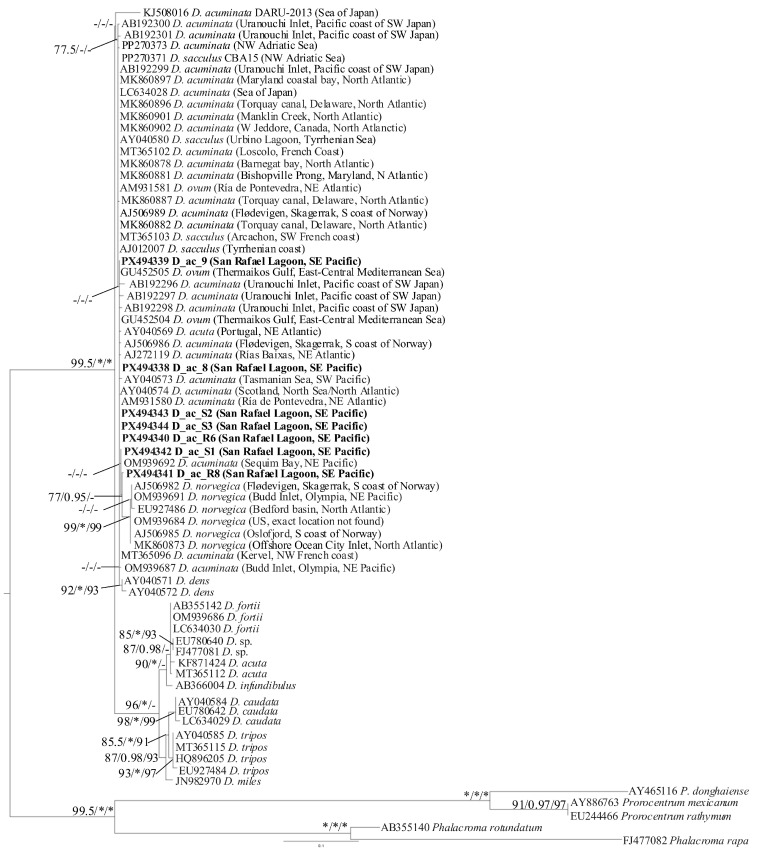
Maximum likelihood phylogenetic tree inferred from ITS rDNA, including sequences of *Dinophysis* species. Three sequences within genus *Prorocentrum* (*P. donghaiense*, *P. mexicanum* and *P. rathymum*) and two sequences within genus *Phalacroma* (*P. rotundatum* and *P. rapa*) were used as outgroups. The ITS sequences of *Dinophysis* cells obtained in this study are in bold. The scale bar represents inferred evolutionary distance in changes/site. Node support represents Shimodaira–Hasegawa approximate likelihood ratio test (SH-aLRT), approximate Bayesian (aBayes) and ultrafast bootstrap (UFBoot) values. Only the values ≥ 70%/≥0.90/≥90% (SH-aLRT/aBayes/UFBoot, respectively) are shown. Asterisks represent maximum statistical support (100%/1/100%).

The ITS rDNA phylogeny (Figure 5) showed the characteristic pattern reported for the genus *Dinophysis*, in which several species form closely related lineages and deeper relationships remain only partially resolved due to the low genetic divergence and extensive rDNA overlap within the group. The *D. acuminata* complex appears as a genetically heterogeneous assemblage that includes sequences identified as *D. acuminata* and *D. norvegica*, with the latter forming a well-supported internal subclade (AJ506982, OM939691, EU927486, OM939684, AJ506985, MK860873).

Notably, additional sequences identified as *D. ovum* (GU452504, AM931581) and *D. sacculus* (MT365103, AJ012007, PP270373) also fall within this complex, reflecting the lack of clear ITS boundaries among members of the *D. acuminata–D. sacculus–D. ovum–D. norvegica* assemblage.

Sequence AY040569, deposited as *D. acuta* but placed within the *D. acuminata* complex clade, displayed 99.4–99.8% identity to the seven *D. acuminata* sequences obtained in this study (only 1–3 nucleotide differences). In contrast, its identity with the other two sequences of *D. acuta* clustered separately from *D. acuminata* complex (KF871424 and MT365112) was markedly lower (92.1–93.4%). These results strongly indicate that AY040569 was misidentified and likely corresponds to a *D. acuminata* specimen rather than to *D. acuta*.

In the ITS rDNA tree, the node with 99/1/99 (SH-aLRT/aBayes/UFBoot) support corresponds to the *D. norvegica* clade. Our sequence PX494341 (identified as *D. acuminata*) does not fall within this group but appears as its immediate sister lineage. This topology reflects the very short genetic distances between *D. acuminata* and *D. norvegica* (Figure 5).

In addition, pairwise comparisons showed that PX494341 shares 96.3–98.6% identity with the *D. norvegica* sequences but a higher identity of 99.6% with both OM939692 and PX494342, which were identified as *D. acuminata*. PX494341 belonged to DNA extracted from five cells corresponding to a small-sized morphotype (27.68 ± 3.41 µm length × 25.90 ± 3.65 µm width), whereas the remaining isolates corresponded to a larger morphotype (43.53 ± 2.32 µm length × 39.03 ± 2.32 µm width). Despite these morphological differences, *D. acuminata* PX494341 grouped with the larger morphotype sequences and exhibited an almost identical nucleotide composition.

### 2.5. Particulate Toxin Analysis

**Figure 6:**
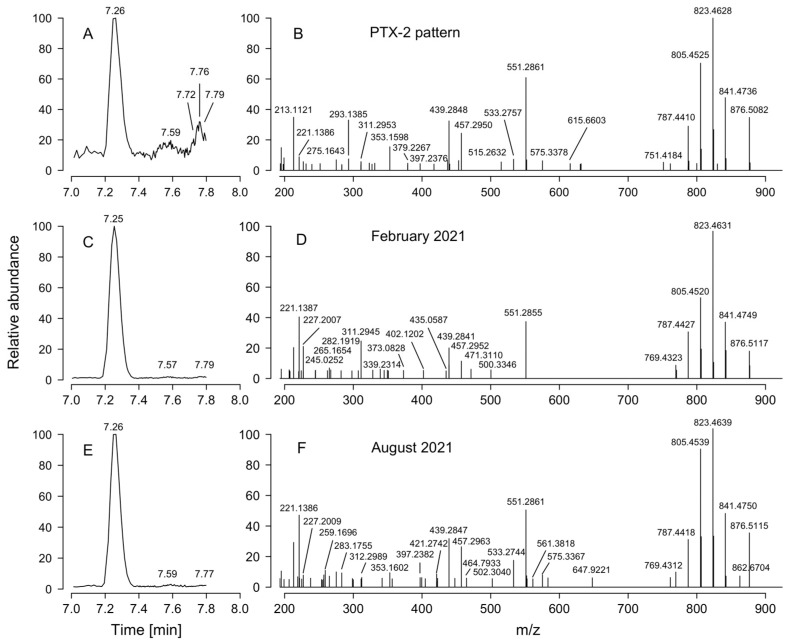
LC-MS chromatograms with their retention times (**left**) and mass spectra (**right**) of pectenotoxin-2 detected in: (**A,B**) PTX-2 standard pattern; (**C**,**D**) net-tow from February 2021 and (**E**,**F**) net-tow from August 2021.

LC-MS analyses of lipophilic toxins in extracts from plankton net-tows collected in summer (February) and winter (August) 2021 revealed the only presence of pectenotoxins (PTX2). A chromatographic peak was detected at 7.26 min with a parent mass [M-H] + 876.5082 m/z which corresponded to PTX2; its presence was confirmed by its characteristic MS/MS fragment at m/z 841.4736, 823.4628, 805.4525 and 787.4401 m/z (Figure 6).

In February 2021, total PTX-2 was 50.2 ng NT^−1^, whereas in August 2021 the total content of the same toxin was slightly lower (35.9 ng NT^−1^). Assuming a fluent (without deflections and/or clogging) passage of seawater through the vertical haul, these values per net-tow would be equivalent to 80 pg PTX2 L^−1^ and 57 pg of particulate PTX2 L^−1^ in February and August, respectively.

## 3. Discussion

*Dinophysis acuminata* is the most widely distributed species of the genus *Dinophysis* and the most frequently cited agent of diarrhetic shellfish poisoning. This work describes a population of *D.* cf *acuminata* which is unique for various reasons: first, its uniform morphology (cell contour) in samples collected in three seasonal surveys (winter, summer and autumn); second, its persistence in moderate cell numbers even during the winter months when the species is under detection limits in most of its usual growing areas. Lastly, it is the only species of *Dinophysis* which seems to be adapted to the location under study. This singular population is reported from a unique place, Laguna San Rafael, a tidewater glacier lagoon with boreal-like waters but located in a temperate latitude and with very stable conditions.

### 3.1. Biogeographic Considerations

*Dinophysis acuminata* is considered a euryhaline and eurythermal species with a capacity to grow under a wide range of salinity and temperature values [40]. This general statement often results from a simple association of its occurrence with physical conditions (temperature, salinity, light) which do not always correspond to those in the depth range where the species grows actively (intrinsic growth rate) or where the population has been entrained and transported (from shelf waters to the coast or vice versa). Furthermore, intraspecific differences in adaptations between strains from different latitudes or systems can be as large as between two different species (Table 1).

**Table 1:** Some examples of *Dinophysis acuminata* strains distributed worldwide. Ranges of temperature and salinity for the occurrence of each particular strain/population and much narrower ranges where high cell densities are reached.

Tolerance		Cell Maximum TS		Location	Geographic Coordinates	References
Temperature (°C)	Salinity	Temperature (°C)	Salinity			
				**Atlantic Europe**		
12.5–22.0	>33.5	13.0–16.0	31–34	NW Spain (Galician Rías)	43° N	[58]
13.6–19.7	32.0–35.4	17.0–18.0	32.0–33.0		42°15′ N; 8°50′ W	[59]
15.0–18.0	32.0–34.4	17.0–18.0	32.0–33.0		42°23′ N; 8°45′ W	[60]
16.0–18.0	<35.5	17.0	35.1		~39°30′–42°30′ N; 09° W	[44]
13.0–18.0	22.0–32.0		23.0–26.0	W Sweden (Fjords)	58°13′ N; 11°33′ E	[61]
15.0–19.0	29.0–34.0	19.0	30.0–33.0	Netherland (N Sea)	~52° N; ~6° E	[62]
				**Baltic Sea**		
7.0–8.0	6.7			NE Sweden	~58° N; ~18° E	[63]
5.5–21.0	5.5–6.9	~18.0	5.5–6.9	NE/NW Finland	~59° N; ~22° E	[64]
5.5–21.0	5.5–6.9	12.0	5.5–6.9			
				**Mediterranean Sea**		
11.0–22.0	29.0–38.0	11.5–12.5	36.0–38.0	E Aegean Sea	~40° N; ~22° E	[65]
				**North Sea**		
7.2–17.1	25.3–34.9	10.4	34.4	Loch Ewe Scotland	57°50′ N; 5°36′ W	[66]
		–		**West Pacific**		
10.0–29.4	22.7–34.0	18.0	28.0	SE Sea Japan	~35° N; ~135° E	[67]
17.3 ± 3.9	32.7 ± 0.85			N Japan	~39° N; ~141° E	[68]
13.0–26.9	27.6–34.1	16.0–18.0	32.0–33.0	SW Japan	33° N; 135° E	[69]
				**NW Atlantic**		
11.1–26.6	17.7–25.2	11.1–26.6	23.8	E USA, New York	40°53′ N; 73° W	[70]
8.7–26.7	8.2–26.7	13.0–24.0			40°53′ N; 73° W	[71]
				**SW Atlantic**		
17.0–19.0	23.0–29.0	~18.0	~25.0	NW Brazil	26°47′ S; 48°37′ W	[72]
5.7–18.4	31.2–34.2	~8.0–13.0		SW Argentine	~38–56° S; 57–69° W	[73]
22.0–26.0	25.0–32.3	26.0	30.2	SW Uruguay	~30–35° S; ~53–58° W	[74]
				**SE Pacific**		
10.5–19.6	4.7–30.73	11.19–12.52	19.75–22.59		43°47′ S; 72°56′ W	[75]
10.2–16.9	17.4–32.1	16.1–16.5	17.4–20.0	N Chilean fjords	44°36′ S; 72°48′ W	[47]
10.8–18.8	8.4–32.0	12.6	31.3		41°36′ S; 72°24′ W	[76]
5.2–8.2	15.0–16.0	8.2	15.0	San Rafael Lagoon	46°40′ S; 73°55′ W	This work
5.8–10.4	23.8–32.5	8.0	30.6	S Chilean fjords	54°49′ S; 70°12′ W	[77]
				**Oceania**		
13.8–17.0	33.8–34.3	14.6–14.8	34.2–34.3	NE New Zealand	~41°20′ S; 174°07′ E	[78]

In a niche-based study of the phytoplankton community in Ría de Vigo, Velasco-Senovilla et al. [79] found that *D. acuminata* is very tolerant and at the same time exhibits high marginality. In other words, it is able to endure a wide range of adverse conditions, including absence of prey up to two months [80], but population growth occurs within a very narrow environmental window [47,81,82,83]. The diverse set of conditions where species of the *D. acuminata* complex bloom (Table 1) suggests there is a variety of strains adapted to endure very different ranges of temperature and salinity. But population increases occur within narrow ranges related to different growth seasons and in response to site-specific triggers. The Baltic Sea shows the lowest temperature and salinity limits and the highest latitude [63,64], but there are reports from unmonitored higher latitude areas visited during ad hoc surveys and scientific expeditions (see “Dana” in Section 3.3). The lower limit for growth (~5 °C) agrees with the temperature identified as the lower limit for *D. acuminata* division in laboratory cultures [84].

### 3.2. Considerations on Morphological Variability in Dinophysis cf acuminata

*D. acuminata* exhibits highly variable morphology. Size and shape of its large hypothecal plates and repartition of the left sulcal lists between daughter cells are affected by the stage in the life cycle (small cells produced by depauperating division) and by the species’ vegetative division by desmoschisis as well as the nutritional status of the cell (a range of sizes from well-fed vacuolate cells to thin starving specimens) [85]. There is also an intraspecific morphological variability which has been explained on the basis of biogeographic differences, but there is controversy on whether these differences are inherited traits or phenotypic responses to environmental conditions which can be stimulated in the laboratory with acclimation experiments [31]. In any case, cosmopolitan species with broad niches present morphological differences between strains growing in different latitudes or hydrodynamic systems. This issue has been well illustrated in the case of species of the genus *Tripos* and the variability (size and shape) of their apical and lateral horns [86].

Among a dozen well-known species of *Dinophysis*, the magnitude of their morphological variability as a source of uncertainty in taxonomic identification has been biased by their toxic potential and the location of the impacted aquaculture sites. The” *Dinophysis acuminata* complex” includes various morphotypes of *D. acuminata*, *D. sacculus* and *D. ovum*, which may have overlapping distributions and are major contributors to DSP events in temperate latitudes. These three taxa cannot be distinguished with conventional rDNA probes applied for species discrimination. Nevertheless, the same or higher uncertainties can be met with other “*Dinophysis* complexes” which include more than one recognized species of *Dinophysis* (e.g., “*D. caudata/D. tripos” and “D. acuminata/D. norvegica”* complexes) and have not drawn much attention.

### 3.3. Taxonomical Assignation and Toxin Profile of Dinophysis from San Rafael Lagoon

*Dinophysis* specimens collected in San Rafael during three different seasons showed a very regular shape. The morphotypes described here, with their coarse and rotund appearance, are reminiscent of some morphotypes described by Paulsen in his section of “Icelandic Dinoflagellates. On Northern *Dinophysis* and *Phalacroma* species” collected in Icelandic fjords (>68° N) during the *Dana* expedition [87].

**Figure 7:**
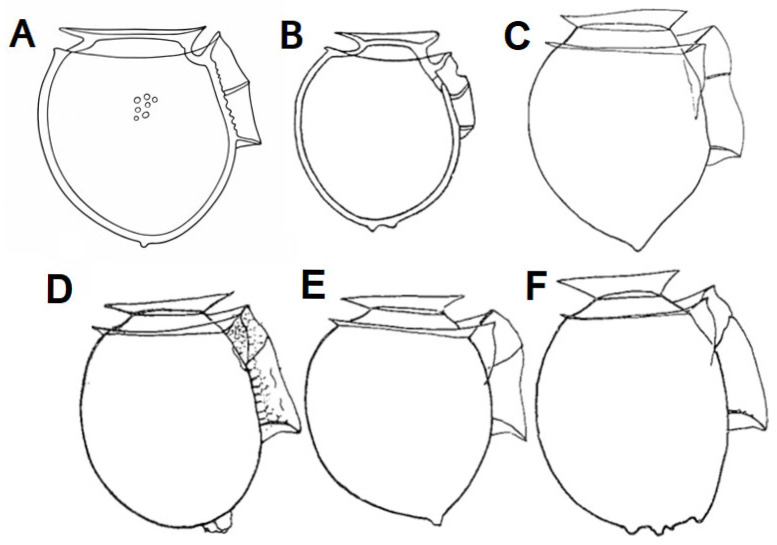
(**A**) *Dinophysis islandica* (modified from Figure 11S in [87]); (**B**) *Dinophysis borealis* (modified from Figure 14U in [87]); (**C**) *Dinophysis norvegica* (modified from Figure 2G in [88]); (**D**–**F**) *Dinophysis acuminata* (modified from Figures 9–11 in [89]).

In a vertical net-haul from Patreks Fjord, Paulsen probably found a multispecific bloom of *D. norvegica*, *D. acuminata* and a few more *Dinophysis* species, each one presenting an array of cell cycle- and life cycle-related forms. The listed taxa included *Dinophysis artica*; *D. islandica*; “Acuminoide *Dinophysis*” (all these with antapical protuberances as in *D. acuminata*, *D. borealis*, *D. lachmanni*), and *D. skagii*, and common descriptive terms for *D. norvegica* included “Atlantic robust forms”, “*pointed*/*obtusa*/*angulata* forms” as well as currently used forms (*debilor*, *crassior*). But different taxa, drawings and proposed names were not equally adopted by the most influential taxonomists. *D. borealis* was included in a group of Norwegian *Dinophysis* species examined (pore theca morphology) by Balech [88], who found them in samples from cold temperate to subarctic latitudes (between Oslofjord and Tromsø) (Figure 7). Balech [88] considered there were no reasons to keep *D. borealis* as a separate species from *D. acuminata* Claparède and Lachmann and considered them to be synonyms. Thus, *D. borealis* corresponds to broad *D. acuminata* morphotypes (L: 39–53, L:D ratio 1.07–1.18) with a quite rounded antapical end, with or without antapical nodes or protuberances. Later he renamed the broad morphotypes, illustrated with the same Norwegian specimen outlines, as *D. acuminata* var *acuminata* [89]. The small size-class observed in summer samples from San Rafael Lagoon is compatible with a small cell formation process during depauperating division of the vegetative cells [90]. These small cells behave as male gametes and form anisogamous mating-gamete pairs with larger cells [49]. Production of small cells seems to be a density-dependent process [79] and is maximal at the peak of annual population growth [91]. Recently, *in situ* imaging technology (Imaging FlowCytobot) has provided evidence of a mass production of small cells in populations of *D. acuminata* aggregated in thin layers preceding mating and formation of planozygotes (2n) with two trailing flagella [92]. This sexual division is segregated in time from the vegetative division at dawn and confirmed early observations by MacKenzie [93] in field populations of *D. acuta* in Marlborough Sound, New Zealand.

*D. acuminata* morphotypes from San Rafael Lagoon are quite different from the more oval and smooth *D. ovum*, a member of the *D. acuminata* complex predominant in high density (>5 × 10^4^ cells L^−1^) blooms reported in southern Brazil [94], Uruguay [74] and off Buenos Aires [95], all these subject to the influence of the plume from La Plata River. They also differ from *D. fortii*, with its characteristic straight ventral margin, misidentified as a member of the *D. acuminata* complex in samples from southeastern Brazil [96]. Lastly, they are clearly distinct from previous observations of *D. acuminata* forms in field samples along the Chilean coasts, including the southernmost Chilean fords in the Magallanes province (Díaz et al. [97] and Table 1), as well as in field and cultured specimens from the Patagonian Pacific coast (off Chiloé Island and outer fjord waters) shown in Paredes-Mella et al. [98].

Phylogenetic analyses of the ITS region clustered the sequences of San Rafael specimens, both small and large morphotypes, with those of *D. acuminata* from North America, western Japan, Australia and Europe and with other morphologically close “species” included in the *D. acuminata* complex (i.e., *D. ovum* and *D. sacculus*). Therefore, the morphological variations observed between the small and large morphotypes and between other forms included in the *D. acuminata* complex do not seem to correspond to genetic differentiation, suggesting they all belong to the same genetic lineage within the *D. acuminata* complex.

Several studies have drawn attention to the lack of an appropriate molecular tool to discriminate between morphologically close species of *Dinophysis*. Sequences of genes used as genetic markers in eukaryotes (i.e., nuclear LSU and SSU rDNA, ITS, and mitochondrial *cox1* and *cob* genes) show little variability between different species of *Dinophysis.* Problems to identify species grouped in the “*D. acuminata* complex continue 35 years after the first mention [99]. But, this is not the only “complex” within the genus *Dinophysis.* Phylogenetic analysis of the ITS rDNA tree here showed *D. norvegica* grouped in a subclade very close to *D. acuminata* as its immediate sister lineage. A very short genetic distance between *D. acuminata* and *D. norvegica* agrees with observations from Edvardsen et al. [100] who concluded that species delineation between *D. acuminata* and *D. norvegica* using morphometrics and genetics was not always apparent and suggested that hybrids existed in Norwegian waters. The coarse texture of the hypothecal plates and broad proportions of *D. acuminata* specimens in the present study make some forms with slightly tapered antapical end look similar to small cells of *D. norvegica* (Figure 7). In fact, some *D. acuminata* images shown in Stern et al. [43] (e.g., Figure 2 Dph2, Dph7, Dph12) seem to be misidentifications corresponding to medium-small sized cells of *D. norvegica* (see *D. norvegica* forma *crassior* in Hansen [101]) and to forms labelled as *D. norvegica* from the Kattegat and published in Larsen and Moestrup [34] as Figure 7D,E. In Loch Ewe (57° 50′ N), a fjord in Northwest Scotland, UK, data available online of cell counts from the official HAB monitoring shows that morphologically close *D. acuminata* and *D. norvegica* are occasionally enumerated together as *D. acuminata*/*D. norvegica* complex https://data.marine.gov.scot/dataset/scottish-coastal-observatory-loch-ewe-site (accessed on 28 January 2026).

Raho et al. [30] proposed sequencing mitochondrial genes as a better marker for *Dinophysis* species. These also failed to resolve the “*acuminata* complex”, but in an extensive phylogeny tree inferred from mitochondrial *cox1* gene sequences (507 bp) of *Dinophysis* prepared by Park et al. [31], *D. norvegica* and *D. acuta* formed individual clades and the other *Dinophysis* species were separated into two heterogeneous clades: the *D. caudata* group, which includes the large pedunculated *Dinophysis* species, and the “*D. acuminata* complex” group.

We are not in a position to make a sound taxonomic assignment of the *Dinophysis* morphotypes. With the available genetic information and cell contour diagnosis, we can confirm that it is a member of the *D. acuminata* complex and close to the broad forms classified as *D. acuminata* var *acuminata* in Balech [88].

Toxin analyses of the net-tows in which *D. acuminata* was the only potentially toxic species showed the presence of PTX2 as the only toxin in the extracts. No okadaates were detected. This simple profile coincides with the predominant PTX2 profile found in most *D. acuminata* populations along the Chilean coast [97] and in several cultivated strains from southern Chile [98]. But *D. acuminata* blooms associated with PTX2 and DTX1 have been occasionally reported in Southern Chile [97,102].

The results of toxin concentration per unit of volume, i.e., PTX2 in ng per net-tow (ng PTXs NT^−1^) have to be taken as semiquantitative information. Quantification of toxins in phytoplankton net-tows and extrapolation to toxin per cell can be biased by multiple factors which were not considered in this work [4]. But they can be compared with results obtained by other experts using the same plankton collection systems and applying the same analytical protocols.

During three cruises in the Argentine Sea (∼38–56° S) in years when *D. acuminata* occurred using the same sampling and analytical procedures [73], maximum values of toxin per net-tow were 624 ng PTX2 NT^−1^ (autumn 2012), 997 ng PTX-2 NT^−1^ (spring 2013) and 44 ng PTX-2 NT^−1^ (late summer 2013). But, the plankton nets used in those cruises had a diameter (60 cm) three times larger than ours (20 cm), so the volume of filtered seawater was 9 times higher and the toxin per unit of volume has to be divided by the same factor. *Mutatus mutandi*, the observed “normalized” concentrations of particulate PTX2 in plankton net tows in the Argentine Sea (2012–2013) were 69 (autumn), 111 (spring) and 5 (summer) ng PTX-2 NT^−1^ *cf*, 50.2 (summer) and 35.9 (winter) ng PTX-2 NT^−1^ in Laguna San Rafael in 2021.

The above estimates suggest moderate levels of PTX2 in San Rafael Lagoon. Unfortunately, the concentration of extracellular toxins was not measured. Nevertheless, a chronic exposure to moderate-low levels of toxic cells (particulate) and their exudates (extracellular toxins) may result in equally noxious effects in sensitive organisms and a relevant accumulation in filter-feeders [103].

### 3.4. Confirmation of Mesodinium rubrum + Major Complex as the Ciliate Prey for D. acuminata in San Rafael Lagoon

*Dinophysis* species are plastidic specialist non-constitutive mixotrophs (pSNCM), i.e., they need to steal plastids from a very specific prey to perform photosynthesis. Identification of the prey is a key issue in the *Dinophysis* population growth equation. Phylogenetic analysis of the (plastid-derived) 23S rDNA sequences confirmed their alignment with the *Teleaulax*/*Geminigera* clade. *Plagioselmis* is now deleted from the clade’s nomination after confirmation that it is the haploid (n) stage in the life cycle of *Teleaulax amphioxeia* [104]. This is the first time that plastids of wild populations of *D. acuminata* from Chile have been sequenced. In an earlier study, Díaz et al. [41] found a different kind of plastid in *D. acuta*, *D. caudata*, *D. tripos* and *D. subcircularis* from Chilean Patagonia. Plastid sequences in picked cells from the four species aligned with those in cryptophytes from clade V (*Rhinomonas*, *Rhodomonas* and *Storeatula*). That study showed a predominance of red cryptophyte plastids different from the *Teleaulax*/*Plagioselmis*/*Geminigera* clade in field populations of phototrophic *Dinophysis* spp., but *D. acuminata* was not included in the occurring species. Previous studies with *Dinophysis* species from northwestern Scotland [43] had reported the first case of phototrophic *Dinophysis* harbouring plastids related to the *Rhodomonas*/*Storeatula* clade.

### 3.5. Singularities of San Rafael Lagoon and Its Dinophysis Populations: Applications for Research and Monitoring

Monitoring and early warning of lipophilic toxin events face several issues derived from the characteristics of the toxin-producing species: (i) detection of low-density populations which may occur for months below detection levels; (ii) difficulties to discriminate between co-occurring *Dinophysis* species with similar morphology but different toxic potential; and (iii) identification of the inoculum source, which is not easy with holoplanktonic species which do not rely on resting cysts as a seeding strategy [36,85]. None of these issues seem to pose a problem in the unique environment of San Rafael Lagoon. Populations of *D. acuminata* were present well above the detection limits with microscopic cell counts using the Utermöhl [57] technique, even during the winter survey. There was a single homogeneous population of *D. acuminata* which did not have to be discriminated from co-occurring morphologically close species of *Dinophysis*. Lastly, the inoculum population was available all the time, and there was a 2–3 doublings difference between the (winter) minimum and the (summer) maximal cell densities observed.

Park et al. [31] illustrated the morphological variation in cultures of 54 strains of *D. acuminata* complex isolated from Korean coastal water. All the original isolates had a *Dinophysis* cf *ovum* appearance, but culture samples taken during the exponential growth phase showed a continuum of shapes from *D. acuminata* to *D. ovum* morphotypes which yielded identical sequences of the mitochondrial cox1 gene. Unfortunately, we have no information on how many days each strain was incubated before the images were taken, and all strains (regardless their isolation site) were grown with identical conditions (20 °C, salinity 30, 14:10 light:dark cycle with cool-white, fluorescent lamps providing a light intensity of 140 μmol photons m^−2^ s^−1^).

Cultured microalgal cells sometimes show important morphological differences (contours become smoother and loose sharpness) compared with wild specimens. These differences increase with days of incubation and are not subject to some field constraints (e.g., turbulence or laminar flows, shear currents, etc.) with important effects on shape but infrequently measured or related to morphological variability. But with no doubt, and until a higher resolution molecular tool is achieved, the results of Park et al. [31] support the view that the “*Dinophysis acuminata* complex” may be the result of phenotypic variants of *D. acuminata* determined by intrinsic factors, such as life cycle, cell cycle stages, nutritional status [85] and extrinsic factors, such as changing environmental conditions and the geographical location [31]. The stable environmental conditions in San Rafael Lagoon and the regular shapes observed in *Dinophysis* cells there give support to the hypothetical relationship between environmental stability and shape (hypothecal plate contours). The closest morphotypes in appearance to the *Dinophysis* from San Rafael Lagoon are the broad sub-Artic forms with coarse hypothecal plates described by Paulsen [87] and Balech [89] in areas with similar low temperatures but saltier waters. In contrast, the illustrated morphotypes from the cold and much fresher Baltic waters [64] agree with the slender forms with a delicate appearance which Balech classified as *D. acuminata* var. *lachmannii.* A possible explanation is that shape and texture in *Dinophysis* are mainly determined by the turbulence regime and that salinity and temperature may be proxies for other hydrodynamics features. Temperature may have an indirect effect on size: cells of the same species from colder areas are usually larger, and a relationship between temperature and division rates (which affects size) has been shown in culture experiments with *Dinophysis* species [84].

The *D. acuminata* population from San Rafael Lagoon contained only pectenotoxins (PTX2). Extracellular PTX2 has been detected with “solid phase adsorption toxin-tracker (SPATT) resins as far south as 62° S in summer, off the South Shetlands (Antartica) (1.5 °C and S = 34) associated with scattered cells of *D. norvegica* and *D. acuminata* [105]. The recent deregulation of pectenotoxins by the European Union Commission [106] has been a relief for shellfish growers exporting bivalves to Europe provided they use current standard LC-MS equipment for their toxin testing. Nevertheless, regions with shellfish resources which continue using mouse bioassay for toxin tests may suffer prolonged false positives and economic losses if their local strains have a simple PTX-predominant toxin profile. Notwithstanding the new scenario concerning *Dinophysis* toxins, growing evidence of the harmful effects of PTXs on early larval stages of marine organisms in vitro needs to be supported by observations in the field.

San Rafael National Park is protected from human activities affecting environmental health, and there is no shellfish exploitation in the lagoon, but a unique wild fauna. A thorough inventory of the local fauna, in particular the recruitment processes in larval stages of fish and invertebrates, would shed light on species sensitivity or tolerance of environmental levels of PTX. In summary, Laguna San Rafael, the only tide-water glacier lagoon remaining in the Northern Patagonian Ice Field, could be used as a privileged natural mesocosm and as a baseline to study the effect of chronic exposure to low levels ofPTXs in marine organisms. Progress could be achieved too on extreme habitat conditions affecting biodiversity and limiting (or selecting) determined species of interest, such as *Dinophysis*, their accompanying species, as well as their competitors.

For all the above reasons, we conclude that what makes San Rafael Lagoon a unique environment is the combination of an Austral temperature regime with a warm-temperate latitude light regime. *Dinophysis* populations and their ciliate prey will have suitable light intensity and day length (photoperiod) for growth in late winter–early spring (time for the spring bloom in southwestern Europe) when there is 24 h darkness in polar latitudes. The eddy circulation reported by Soto et al. [107] may act as a retention area and explain the maintenance of moderate overwintering cell densities of *Dinophysis*. The extreme conditions of the lagoon, at the extreme latitude of existing glaciers, will act as a filter of stress-tolerant organisms and promote low diversity communities. It may also explain the absence of *D. norvegica*, co-occuring with *D. acuminata* in any other cold-temperate, boreal/austral system mentioned in Table 1.

### 3.6. Future Research Directions

The study of Davenport in the early 1990s [56] is the only information available on the phytoplankton communities in the lagoon. Sampling in that study was carried out with horizontal hauls of plankton nets of 40 µm mesh size in a single survey in summer. But the author pointed to the very low diversity (a bloom of *Coscinodiscus*) and the homogeneous composition of the phytoplankton communities throughout a 24 h cycle.

There are many questions related to *Dinophysis* population dynamics and early warning systems (EWS) which can be elegantly tested in Laguna San Rafael, a natural observatory extremely sensitive to climate change effects on calving glaciers.

There are ongoing research projects on the lagoon circulation and connectivity with adjacent water bodies, as well as on climate change-driven changes in the calving glacier’s limits. The challenge for HAB experts is to elucidate the hypothetical role of San Rafael Lagoon as a *Dinophysis* retention area for overwintering populations, or as a *Dinophysis* incubator and pelagic seed bank ready to export seed populations to the neighbouring fjords and channels. Alternatively, the lagoon may be an isolated body of water with connections limited to a thin surface layer. This last possibility would justify the morphology of a *Dinophysis* strain distinct from other populations in fjords and inner seas in Southern Chile.

## 4. Materials and Methods

### 4.1. Study Area

The Chilean Patagonian coast (southeastern Pacific Ocean), bordered to the east by the Andes Mountain Range, is one of the most extensive (from 41.5° S to 56.7° S) and complex fjord and channel systems in the world. Northern Chilean Patagonia extends from Puerto Montt (41.5° S) in the Los Lagos Province to the Taitao Peninsula (46.5° S) in Aysén [108]. The Northern Patagonian Icefield (NPI) caps the Andes Mountain range throughout the Northern Patagonia limits, and San Rafael Glacier is in its southernmost sector (Figure 1). San Rafael Glacier, one of 39 calving glaciers in the NPI, discharges into San Rafael Lagoon [52,53,109]. San Rafael Lagoon (46°40′ S; 74°56′ W) is the only tidewater glacier lagoon remaining in the Northern Patagonian Icefield and the lowest latitude tidewater glacier in the world. This fan-shaped (15 km long, 10 km wide) body of water has a maximal depth of 250 m in the area close to the glacier and temperatures ranging from 5.5 to 6.5 °C [110]. The lagoon is connected to the Pacific Ocean through “Río Témpanos” (=“Ice Floe River”, not really a river but a channel about 15 m deep) and Golfo Elefantes (Elephants Gulf), an elongated fjord.

Soto et al. [107] developed a high-resolution hydrodynamic model which described an anticyclonic eddy in the central part of the lagoon. The sampling station in this work, in the middle point of this eddy, was chosen as representative of the main central area of San Rafael Lagoon which may act as a retention area for phytoplankton populations.

*Dinophysis* specimens described here are from opportunistic samples collected with oceanographic bottles and vertical net-tows in Laguna San Rafael during cruises aimed to study the hydrodynamics and connectivity of the lagoon with adjacent water bodies. (Figure 1).

### 4.2. Sampling Overview

Between November 2020 and August 2021, three oceanographic cruises (15 November 2020; 25 February and 5 August 2021) were carried out in a southern section (46°20′–46°40′ S) of NW Patagonia on board *R.V. Dr. Jürgen Winter.* On each cruise, opportunistic sampling was carried out in one station (Figure 1, white dot on San Rafael Lagoon) to collect bottle samples for phytoplankton observations.

During each visit to the station, vertical profiles of temperature, salinity and *in vivo* chlorophyll *a* fluorescence were obtained with an AML (AML Oceanographic, Victoria, BC, Canada) CTD profiler model Metrec-XL equipped with a Turner Designs CYCLOPS-7 (Turner Designs, San Jose, CA, USA) submersible fluorometer (excitation 460 nm, emission, 620–715 nm).

Samples for quantitative analyses of micro-phytoplankton were collected with Niskin bottles at six discrete depths from surface to 50 m depth (0, 5, 10, 15, 20 and 50 m). Subsamples of 100 mL were collected in opaque glass jars and immediately fixed with acidic Lugol’s solution [111]. Samples for semiquantitative analysis of toxins in the plankton (toxin content per litre) were collected with vertical net-tows (20 μm mesh Nitex, 20 cm Ø,) through the upper 20 m of the water column; the filtered volume per vertical tow was about 628 L [73]. Samples were reduced to a final volume of 50 mL (adding filtered seawater if needed) and filtered through Whatman GF/F fiberglass filters (25 mm Ø, 0.7 μm pore size) (Whatman, Maidstone, UK). The filters and filtered material were placed in a cryotube, mixed with 1 mL analysis grade methanol and stored in the laboratory at −20 °C until analysis.

### 4.3. Phytoplankton Analyses

For quantitative analyses of phytoplankton, 10 mL of the Lugol’s fixed bottle samples were left to sediment overnight and analyzed under an inverted Olympus CKX41 microscope (Olympus, Tokyo, Japan) using the method described in Utermöhl [57]. Large but less abundant taxa, such as *Dinophysis,* were counted at a magnification of ×100, so that the detection limit was 100 cells L^−1^. Confidence limits of the cell counts were calculated according to Andersen and Throndsen [112].

### 4.4. Dinophysis Morphometry and Imaging

Observations were conducted on acidic Lugol’s fixed *Dinophysis* cells at ×20 magnification using a Nikon Eclipse TS2 inverted microscope (Nikon Corporation, Tokyo, Japan), equipped with an industrial-grade digital microscopy camera (model X7CAM4K16MPA) with 4K full-HD resolution and autofocus capabilities, enabling high-quality image acquisition under bright-field illumination. Maximum cell length (L) and dorso-ventral depth (D) of the large hypothecal plates were measured following the criteria of Balech [88]. Measurements were performed on digital images acquired using ToupTek XCamView software (version V1.8_20240530) and subsequently processed and analyzed with the free software ImageJ (version 1.54i) [113]. A total of 44 normal-sized cells and 9 small forms of *Dinophysis* were measured.

### 4.5. Single Cell Isolation, PCR Amplification and Sequencing

Cells of *Dinophysis* from the acidic Lugol’s preserved samples collected in Laguna San Rafael were individually picked with a capillary pipette and washed three times in drops of distilled water on a glass slide. Samples were photographed and measured with a Nikon Eclipse TS2 equipped with a 4K digital camera and transferred to 200 µL tubes. Then, a cold shock with liquid nitrogen was applied and samples were kept at −80 °C before immediate PCR analyses.

After a series of unsuccessful assays on single cells, tubes containing between 4 and 33 specimens showed positive results. ITS (ITS1-5.8S-ITS2) and partial plastid 23S rRNA gene were amplified using the pairs of primers ITSF01/PERK-ITS-AS (5′-TCCCTGCCCTTTGTACACAC-3′/5′-GCTTACTTATATGCTTAAATTCAG-3′, [114]) and p23Sr_f1/23Sr_r1 (5′-GGACAGAAAGACCCTATGAA-3′/5′-TCAGCCTGTTATCCCTAGAG-3′, [115]). The amplification reaction mixtures (20 mL) were performed using Horse-Power™ Taq DNA Polymerase MasterMix (2x) (Canvax, Valladolid, Spain). DNA was amplified in an Eppendorf Mastercycler EP5345 (Eppendorf AG, New York, NY, USA). PCR conditions were as follows: initial denaturation for 5 min at 94 °C, followed by 35 cycles of 35s at 94C, 35 s at 54 °C (ITSF01/PERK-ITS-AS), 55 °C (p23Sr_f1/23Sr_r1), and 1 min at 72 °C, followed by a final extension for 7 min at 72 °C. An 8 µL aliquot of each PCR reaction was checked by agarose gel electrophoresis (1% TAE, 50 V) and GelRed™ nucleic acid gel staining (Biotium, Hayward, CA, USA). PCR products were purified with ExoSAP-IT™ (USB Corporation, Cleveland, OH, USA). Sequencing reactions were performed using the Big Dye Terminator v3.1 reaction cycle sequencing kit and migrated in a SeqStudio genetic analyser (both at Applied Biosystems, Foster City, CA, USA) at the CACTI sequencing facilities (Universidade de Vigo). The ITS and plastid 23S rDNA sequences obtained in this study were deposited in GenBank (ITS Acc. Numbers are *Dinophysis*_ac_8 PX494338, *Dinophysis*_ac_9 PX494339, *Dinophysis*_ac_R6 PX494340, *Dinophysis*_ac_R8 PX494341, *Dinophysis*_ac_S1 PX494342, *Dinophysis*_ac_S2 PX494343 and Dinophysis_ac_S3 PX494344 and plastid 23S Acc. Numbers are PX498283 and PX498284 for 2_23S_San_Rafael_Dinophysis and 2_23S_San_Rafael_Dinophysis).

### 4.6. Alignment and Phylogenetic Analyses

Plastid 23S and internal transcribed spacer (ITS) rDNA sequences were analyzed separately. Each group of sequences was inspected, aligned and trimmed using Geneious Prime^®^ 2024.0.7 (Biomatters Ltd., London, UK). Each alignment was performed using MAFFT v7 [116] with the E-INS-i algorithm, and pairwise identity (%) matrix was calculated with Geneious Prime^®^ 2024.0.7 (Biomatters Ltd., London, UK). Patristic distances (measured in substitutions per site) were calculated using the software PATRISTIC v1.0 [117] and represented the sum of branch lengths between pairs of taxa in the phylogenetic tree. This distance was used to quantify evolutionary divergence among sequences.

Phylogenetic analyses were conducted in IQ-TREE v3.0.1 [118]. The best-fitting nucleotide substitution model was determined automatically using ModelFinder [119] according to the Bayesian Information Criterion (BIC), resulting in selection of the TPM3v + G4 model in the case of the 23S alignment and TIM2 + F + G4 for the ITS. The phylogenetic trees were represented using the ML method. Node support was assessed using three complementary methods: Shimodaira–Hasegawa approximate likelihood ratio test (SH-aLRT; 1000 replicates) [120], approximate Bayesian (aBayes) support [121] and ultrafast bootstrap (UFBoot; 1000 replicates) [122]. The final consensus maximum-likelihood tree was visualized in FigTree v1.4.4 [123], displaying all three support values at each node (SH-aLRT/aBayes/UFBoot). Following standard practice, nodes with SH-aLRT ≥ 80%, aBayes ≥ 0.95 and UFBoot ≥ 95%, were considered to have strong statistical support, whereas values between SH-aLRT 70–79%, aBayes 0.90–0.94 or UFBoot 90–94% indicated moderate support. Nodes below these thresholds were regarded as weakly supported.

### 4.7. Toxin Analysis

#### 4.7.1. Sample Extraction

The filters and filtered net-towed material kept with methanol in cryotubes were thawed and concentrated by centrifugation (10,000 *g*; 20 min) to obtain a final volume of 1 mL. Each sample was then mixed with 1 mL of methanol (100%) and sonicated with a Branson Sonic Power 250 disruptor cellular sonifier (Thermo Fisher Scientific, San Jose, CA, USA) to extract lipophilic toxins. The extract obtained was clarified by centrifugation (10,000 *g*; 20 min), and the supernatant was then filtered through a 0.20 μm Clarinert nylon syringe filter (13 mm Ø) (MZ-Analysentechnik GmbH, Mainz, Germany). To analyze free OA, DTX1, PTX2, PTX2sa, YTX, and other lipophilic toxins, an aliquot of the sample (0.5 mL) was placed in an amber vial and kept at −20 °C until analysis. To analyze total OA, samples were subjected to alkaline hydrolysis following the standard procedure of the EU Reference Laboratory for Marine Biotoxins [124].

#### 4.7.2. Toxin Detection and Quantification

The presence of lipophilic toxins in the extracts was checked by UHPLC-MS/MS using a Dionex Ultimate 3000 chromatographic system (UHPLC) (Thermo Fisher Scientific, San Jose, CA, USA) coupled to a Thermo Q Exactive Focus by means of a HESI-II electrospray interface, following the method described by Regueiro et al. [125], but modified in order to use a shorter column and to allow enough time for the elution of the toxins. Chromatographic separation was performed using a column Gemini NX-C18 (50 mm × 2 mm; 3 μm) from Phenomenex (Torrance, CA, USA). The flow rate was set to 0.35 mL min^−1^, and the injection volume was 10 μL. The mobile phase was used in gradient mode as follows: 85% of eluent A (100% water containing 6.7 mM NH_4_OH) and 15% of eluent B (90% acetonitrile:10% water with 6.7 mM NH_4_OH) was held for 1 min, followed by a linear increase to 80% B for 2.85 min and then an increase to 85% B for 0.15 min, and 90% B for 0.75 min and 100% B for 3.25 min. Finally, the gradient was returned to the initial conditions over 2 min and the column was re-equilibrated for 1 min. The mass spectrometer was operated using positive and negative acquisition experiments with the following settings: spray voltage positive 3500 V, negative 3000 V, sheath gas pressure 30, auxiliary gas flow 4, capillary temperature 350 °C, automatic gain control (AGC) of the C-Trap 5 × 10^4^, and mass resolution of 70,000 FWHM.

For quantification and confirmation, data acquisition was performed using selected-ion-monitoring (SIM) with data-dependent MS/MS (ddMS^2^). All analyses were performed using a mass inclusion list, including the precursor ion masses, expected retention time window, and collision energy (CE) for each toxin (Table 2). The toxin concentrations of OA, DTX-1, PTX-2, PTX-2sa, and YTX in the extracts were quantified by comparing the areas or peaks obtained in the chromatograms with those of certified reference materials obtained from NCR, Canada. The detection limits of the analyses (LOD) were 0.32 ± 0.03 ng mL^−1^ for OA; 0.24 ± 0.02 ng mL^−1^ for DTX1; and 2.61 ± 0.25 ng mL^−1^ for PTX2. Limits of quantification (LOQ) were 0.50 ± 0.03 ng mL^−1^ for OA; 0.50 ± 0.03 for DTX1; and 4.33 ± 0.25 for PTX2.

The total amount of toxins per sample extract corresponded to vertical net-tows and was expressed as ng of toxin per net-tow (ng NT^−1^) as in Fabro et al. [73]. The net-tow estimate was divided by the volume of filtered seawater during the vertical tow, and the toxins were expressed as pg toxin L^−1^. Considering the fact that the seawater passing through the net may suffer deflections, and also that the mesh can get clogged and thus may not be able to be filter efficiently, data obtained here must be considered as semiquantitative.

**Table 2:** Ultra High Resolution Mass Spectrometry (UHRMS) analysis parameters for the target compounds.

Toxin	Abbreviation	Molecular Formula	Diagnostic Ion	Calculated *m*/*z*	CE
Okadaic acid	OA	C_44_H_68_O_13_	[M − H]^−^	803.4587	55
Dinophysistoxin-1	DTX-1	C_45_H_70_O_13_	[M − H]^−^	817.4744	55
Dinophysistoxin-2	DTX-2	C_44_H_68_O_13_	[M − H]^−^	803.4587	55
Yessotoxin	YTX	C_55_H_82_O_21_S_2_	[M − 2H]^2−^	570.2322	34
homo-Yessotoxin	homoYTX	C_56_H_84_O_21_S_2_	[M − 2H]^2−^	577.2401	34
Azaspiracid-1	AZA-1	C_47_H_71_NO_12_	[M + H]^+^	842.5049	34
Azaspiracid-2	AZA-2	C_48_H_73_NO_12_	[M + H]^+^	856.5206	34
Azaspiracid-3	AZA-3	C_46_H_69_NO_12_	[M + H]^+^	828.4893	40
Gymnodimine	GYM	C_32_H_45_NO_7_	[M + H]^+^	508.3421	34
Pinnatoxin-G	PnTX	C_42_H_63_NO_7_	[M + H]^+^	694.4677	50
13-desMe-SPX C	SPX-1	C_42_H_61_NO_7_	[M + H]^+^	692.4521	34
Pectenotoxin-2	PTX2	C_47_H_70_O_14_	[M + H_2_O]^+^	876.5104	30

## 5. Conclusions

Opportunistic samplings revealed the occurrence of *Dinophysis* cf *acuminata* in a tidewater glacier lagoon formed by the climate change-driven retreat of San Rafael Glacier, in San Rafael Lagoon National Park and Reserve of the Biosphere.

The broad and coarse morphotypes of *Dinophysis* found in San Rafael Lagoon, with a toxin profile of pectenotoxins (PTX2) only, are similar to the description of Paulsen’s [87] broad and semi-circular (L:D = 1.18) forms first reported in Boreal waters (northwestern Iceland) and considered by Balech [89] to be synonyms of *D. acuminata* var. *acuminata*.

Partial sequences of ITS rDNA aligned *D. acuminata* from San Rafael Lagoon with another member of the *D. acuminata* complex from North America, Europe and Japan. Partial sequences of their plastid derived 23S rDNA, the first plastid sequence obtained from field specimens of *D. acuminata* in Chile, confirmed ciliates of the *Mesodinium rubrum* + *major* complex as their prey and plastid source.

The detection of moderate (>500 cells L^−1^) cell densities of *Dinophysis* cf *acuminata* in San Rafael Lagoon as well as the regular shape of the cells in the three seasonal (spring, summer and winter) surveys appear associated with extreme but stable conditions of a lagoon with an Austral temperature regime and a warm-temperate latitude light regime. These features added to the occurrence of one single species of *Dinophysis* with a simple toxin profile makes the lagoon a natural observatory for ecophysiology, ecotoxicology and population dynamic studies with a focus on the *Dinophysis acuminata* complex in a highly sensitive environment to climate change.

## Data Availability

The original contributions presented in this study are included in the article. Further inquiries can be directed to the corresponding author.
